# Chromium exposure and incidence of metabolic syndrome among American young adults over a 23-year follow-up: the CARDIA Trace Element Study

**DOI:** 10.1038/srep15606

**Published:** 2015-10-22

**Authors:** Jianling Bai, Pengcheng Xun, Steve Morris, David R. Jacobs, Kiang Liu, Ka He

**Affiliations:** 1 Department of Epidemiology and Biostatistics, School of Public Health—Bloomington, Indiana University, Bloomington, IN, USA; 2 Department of Epidemiology and Biostatistics, School of Public Health, Nanjing Medical University, Jiangsu, China; 3 Research Reactor Center, University of Missouri-Columbia and Harry S Truman Memorial Veterans Hospital, Columbia, MO, USA; 4 Department of Epidemiology, School of Public Health, University of Minnesota, Minneapolis, MN, USA; 5 Department of Preventive Medicine, Feinberg School of Medicine, Northwestern University, Chicago, IL, USA

## Abstract

Studies suggest that chromium deficiency is associated with elevated levels of fasting blood glucose, circulating insulin, cholesterol and triglycerides, and decreased proportion of lean body mass. However, data directly relating chromium levels to metabolic syndrome (MetS) risk are lacking. A total of 3,648 American adults from the Coronary Artery Risk Development in Young Adults (CARDIA) study, aged 20–32 years, were prospectively examined for the incidence of MetS and its five components from 1987–88 to 2010–11. Baseline toenail chromium levels were measured with instrumental neutron-activation analysis. Incident MetS was defined by the NCEP-ATP III criteria. During the 23-year follow-up, 878 incident MetS cases were identified. Baseline toenail chromium was inversely associated with incidence of MetS as well as its blood lipid components. The multivariable-adjusted hazard ratio (HR) (95% confidence interval [CI]) of MetS comparing the highest to the lowest quartiles of toenail chromium levels was 0.80 (0.66–0.98; *P*_linear trend_ = 0.006). The adjusted HRs were 0.82 (0.68–0.98; *P*_trend_ = 0.045) for having abnormal triglycerides levels and 0.75 (0.64–0.88; *P*_trend_  = 0.030) for having abnormal HDL cholesterol levels. Toenail chromium levels were inversely and longitudinally associated with incidence of MetS in American young adults. This inverse association was mainly explained by its relation to blood lipids.

Metabolic syndrome (MetS), a cluster of risk factors of cardiovascular disease (CVD) and type 2 diabetes mellitus^1^,^2^, has become a major public health challenge. The age-adjusted prevalence of MetS in American adults was 22.9% in 2009–2010 based on the National Health and Nutrition Examination Survey (NHANES)^3^. Chromium is an essential trace element required for carbohydrate, lipid and protein metabolism^4^. It occurs in the environment primarily in two valence states, trivalent chromium (an essential element in humans) and hexavalent chromium (a harmful element to humans). Two case-control studies^5^,^6^ found an inverse association of toenail chromium levels with the risk of myocardial infarction (MI). Also, laboratory studies have suggested that chromium deficiency was associated with elevated levels of fasting blood glucose^7^, insulin^8^, cholesterol^9^, triglycerides^10^, and reduced levels of high density lipoprotein cholesterol (HDL)^10^. However, data directly relating chromium exposure to the risk of MetS are lacking. Therefore, we prospectively examined baseline toenail chromium levels in relation to incidence of MetS in a large cohort of American young adults over 23 years of follow-up, the Coronary Artery Risk Development in Young Adults (CARDIA) Study.

## Methods

### Study Design

The CARDIA Study is a longitudinal investigation of CVD risk factors in 5,115 men and women aged 18 to 30 years at study inception from 1985 to 1986. Details of the study design, recruitment, and procedures have been published elsewhere^11^. Briefly, participants were recruited from 4 US clinical sites: Birmingham, Alabama; Chicago, Illinois; Minneapolis, Minnesota; and Oakland, California. The sample was roughly balanced at baseline within center by age (18–24, 25–30 years), sex, race (African American, Caucasian), and education level (high school graduate or less, greater than high school). Participants were reexamined at 2, 5, 7, 10, 15, 20, and 25 years after inception, with retention rates of 91%, 86%, 81%, 79%, 74%, 72%, and 72% at respective follow-up exams. All participants signed informed consent forms, with all aspects reviewed and approved by the institutional review board of each participating institution. And all the methods were carried out in accordance with the approved guidelines.

Among 4,362 participants who provided toenail clippings at baseline in 1987, we excluded those without data on toenail chromium level (*n* = 18). Then, we excluded 169 participants with insufficient information for defining incident MetS during follow-up, 146 with prevalent MetS at baseline, 169 with missing data on individual components of MetS at baseline, and 24 with no information on key covariates (BMI, education, family history of diabetes, smoking status, alcohol consumption, physical activity, and intakes of long-chain n-3 polyunsaturated fatty acid (LCn-3PUFA) and magnesium). In addition, 188 women were excluded because of pregnancy during the follow-up. A total of 3,648 participants remained in the analysis after these exclusions.

### Assessment of toenail chromium

Toenail clippings were collected with a stainless-steel clipper from all 10 toes by the participants themselves during the clinical examination or at home in 1987 and stored at ambient room temperature and humidity. In the laboratory, the clippings were broken into small pieces and cleaned by sonication in 10% nitric acid followed by deionized water. The cleaned samples were collected on a filter and washed thoroughly with deionized water, freeze-dried, and gravimetrically transferred to high-purity quartz vials that were then vacuum-heat-sealed. The toenail chromium concentrations were assessed by instrumental neutron-activation analysis (INAA) at the University of Missouri-Columbia Research Reactor Center^12^. The samples were assayed in random order by laboratory personnel blinded to the disease status and other clinical measures.

The limit of detection is 0.01 μg/g with a sample toenail mass of at least 25 mg, and it increases to 0.1 μg/g when the sample mass decreases to 5 mg or less. We analyzed replicates of 2 different quality control samples for chromium with every batch of toenails. The high-concentration quality control (QC) is NIST SRM 1571 Orchard Leaves that has a certified Cr concentration of 2.6 ± 0.3 μg/g and was measured at 2.8 ± 0.3 μg/g. The low-concentration QC is a ground hair sample that has a chromium concentration of 0.167 ± 0.022 μg/g and was measured at 0.162 ± 0.022 μg/g.

Toenail chromium levels are recognized as good biomarkers of integrated exposure from diet and environment^13^, and have been used in epidemiological studies^14^,^15^. A study found that the Spearman correlation coefficient for the reproducibility of toenail levels of chromium was 0.33 over 6 years among 127 US women^16^.

### Ascertainment of Metabolic Syndrome and Insulin Resistance

Fasting glucose was measured by the hexolinase method^17^. Triglyceride was assessed by enzymatic methods^18^, and HDL cholesterol was quantified by dextran sulfate–magnesium precipitation^19^. Blood pressure (BP) was measured using a random-zero sphygmomanometer before year 20 and the *OmROn* HEM907XL recalibrated to the random zero standard thereafter by trained and certified technicians^20^. BP measurements were taken three times on the right arm with the participant seated at 1-minute intervals after 5 minutes of rest. The average of the second and third measurements was used for the analyses.

MetS was determined at each exam following the National Cholesterol Education Program/Adult Treatment Panel III (ATP-III) definition^21^. An incident event was identified if participants developed ≥3 of the following at any exam after year 2 among those who did not meet this criterion at baseline: fasting glucose level ≥6.1 mmol/L; systolic blood pressure ≥130 or diastolic blood pressure ≥85 mm Hg; waist circumference >102 cm for men or >88 cm for women; triglyceride level ≥1.70 mmol/L; HDL cholesterol level <1.04 mmol/L in men or <1.30 mmol/L in women. Participants who reported using anti-diabetic or anti-hypertensive medications were classified as having high glucose or high blood pressure. Fasting glucose levels were coded as missing if participants had a fasting time <8 hours before the examination. Details of measurement of fasting plasma insulin and calculation of homeostasis model assessment of insulin resistance (HOMA-IR) and HOMA of β-cell function have been described previously^22^.

### Assessment of covariates

Demographic variables, including age, sex, ethnicity and education level, were collected through a self-administered questionnaire and verified during clinic examinations. Body weight and height were measured in light clothes without shoes during the clinical examination. BMI was calculated as weight in kilograms divided by the square of height in meters. Concurrent smoking status was determined based on self-report, and participants were classified into three groups: never smokers, former smokers, and current smokers. Alcohol consumption was measured by a validated questionnaire and classified into four groups according to total daily intake: 0 (never drink), 0.1–9.9, 10.0–19.9, or ≥20 g/day. Physical activity was assessed using the CARDIA Physical Activity History Questionnaire^23^. The physical activity score was calculated in exercise units (EU) reflecting the frequency and duration of activity over the previous year. A score of 100 EU is roughly equivalent to participation in a vigorous activity 2 to 3 hours/week for 6 months of the year^24^. Family history of diabetes was defined as either the mother or father having diabetes.

### Statistical analysis

CARDIA exam year 2 (1987–1988) was set as the baseline of the present study because toenail clippings were collected at that time. Each participant contributed person-time from baseline to the examination date when incident MetS was identified, censored or end of the study at exam year 25, whichever came first. In the analyses, participants were divided into quartiles based on the distribution of toenail chromium levels. Means ± standard deviations and percentages were used to describe characteristics of the study population for continuous and categorical variables, respectively. Group comparisons were made using analysis of variance (ANOVA) or chi-squared test as appropriate.

Cox proportional hazards models were used to assess the association of toenail chromium levels with incidence of MetS by calculating multivariable-adjusted hazard ratios (HRs) and 95% confidence intervals (CIs). The proportional hazards assumption was verified by adding time and chromium interaction terms in the Cox model. We considered three sequential models in the main analysis: model 1 adjusted for age, sex, ethnicity, and study center; model 2 additionally adjusted for BMI, education, family history of diabetes, smoking status, alcohol consumption, physical activity, and intakes of long chain n-3 polyunsaturated fatty acid fatty acids (LCn-3PUFAs) and magnesium; and model 3 further adjusted for the potential effects of baseline status of individual components of MetS. These baseline individual components were categorized based on clinically meaningful cut-offs: SBP (<120, 120 ~ 139, ≥140, mmgHg), glucose (<100, 100 ~ 125, ≥126, mg/dL), triglyceride level (<1.70, 1.70~2.25, ≥2.26, mmol/L), waist circumference (male: <102, ≥102; female: <88, ≥88, cm), HDL (male: <1.04, ≥1.04; female: <1.30, ≥1.30, mmol/L). A continuous variable of toenail chromium level was used for testing the linear trend.

We also investigated possible interactions between toenail chromium and pre-identified potential effect modifiers by adding a corresponding multiplicative interaction term in the models, followed by the likelihood ratio test. In addition, to explore potential mechanisms, we examined toenail chromium levels in relation to fasting insulin, HOMA-IR, and HOMA of β-cell function with available data. Since these variables were measured repeatedly, generalized estimating equations with exchangeable correlation structure were used.

All data management and analyses were performed using SAS (version 9.3; SAS Institute, Cary, NC, USA). *P* ≤ 0.05 was considered statistically significant.

## Results

Among the 3,648 participants included in the longitudinal analyses, median toenail chromium levels across quartiles were 0.2, 0.4, 0.8, and 3.5 μg/g, respectively. Compared with those in the lowest quartile of toenail chromium, participants in the highest quartile were slightly younger, more likely to be females and Caucasians, exercised more, and had a slightly higher education level. They also had lower levels of triglycerides, a lower systolic blood pressure, and higher HDL cholesterol levels (Table 1).

A total of 878 incident cases of MetS were identified during 23 years of follow up. After adjustment for potential confounding variables, a statistically significant graded inverse association between toenail chromium concentrations and incidence of MetS was found. Comparing the highest to the lowest quartile, the HR (95% CI) was 0.74 (0.61–0.90; *P* for trend = 0.003). The inverse association persisted after further adjustment for the baseline levels of individual components of MetS (HR: 0.80; 95% CI: 0.66–0.98; *P* for trend = 0.006) (Table 2).

For the 5 components of MetS, the identified incident events numbered 654 for meeting the glucose criterion, 1,446 for blood pressure, 1,360 for waist circumference, 1,024 for triglycerides, and 1,284 for HDL cholesterol. Significant inverse relations were observed between toenail chromium and triglycerides and HDL cholesterol. Comparing the highest to the lowest quartile of toenail chromium levels, the adjusted HRs (95% CI) were 0.82 (95% CI: 0.68–0.98; *P* for trend = 0.045) for elevated triglycerides levels and 0.75 (95% CI: 0.64–0.88; *P* for trend = 0.030) for having abnormal HDL cholesterol levels. For other individual components, results were not statistically significant (Table 3).

In the subgroup analyses, we examined a few pre-specified potential effect modifiers. The observed inverse association between toenail chromium levels and incidence of MetS was not appreciably modified by sex, ethnicity (African American or Caucasian), baseline BMI (normal weight or overweight), or baseline HOMA-IR score (≥2.5 or <2.5 mU/L × mmol/L). All tests for interaction were statically non-significant.

In the sensitivity analysis, we excluded two lipid components, i.e., triglycerides and HDL cholesterol, when defining the incident cases of MetS. The association between chromium levels and incidence of MetS no longer persisted (data not shown). In addition, the results were not appreciably changed when coding pregnant women at any examination as missing instead of completely excluding them in the analysis.

To explore potential mechanisms, we investigated the association between toenail chromium concentrations and repeated measurements of fasting insulin and HOMA-IR, as well as HOMA of β-cell function index, excluding those participants who had fasting time less than 8 hours. A significant inverse association was found between toenail chromium and HOMA-IR (*β* = −0.0177; 95% CI: −0.0343,−0.0012; *P* for linear trend = 0.036) and a marginal significant inverse association was found with fasting insulin (*β* = −0.0142; 95% CI: −0.0287–0.0004; *P* for linear trend = 0.056). No association was found between toenail chromium and HOMA of β-cell function index (data not shown).

## Discussion

In this prospective cohort study, we found that toenail chromium concentrations were inversely and longitudinally associated with the incidence of MetS among American young adults independent of the baseline levels of MetS components. This inverse association was mainly explained by the relations between toenail chromium concentration and the lipid profile, particularly, the triglycerides and HDL cholesterol levels. These observed associations were not appreciably modified by sex, race, or baseline BMI.

To the best of our knowledge, no longitudinal study or clinical trial has been reported on the association between toenail chromium and incidence of MetS. Two case-control studies suggested an inverse association of toenail chromium levels with the risk of MI^5^,^6^. One cross-sectional analysis using the data from the Health Professionals Follow-up Study suggested that diabetic men with CVD had lower toenail chromium levels than healthy controls^15^.

Although data are not entirely consistent, accumulating evidence from both experimental and observational studies suggests that chromium may have beneficial effects on individual components of MetS. Numerous studies indicate that chromium is essential for lipid metabolism. Animal studies suggest that chromium treatment is associated with a reduction in liver triglyceride levels and lipid accumulation^25^. Also, rats and rabbits fed a chromium deficient diet had elevated total cholesterol and aortal lipid concentrations^26^,^27^. In addition, increased HDL-cholesterol levels^28^ and decreased total cholesterol, LDL-cholesterol and triglycerides levels^29^ have been observed in humans after chromium supplementation. The possible explanations are that chromium may improve the conversion of acetyl coenzyme A (acetyl-CoA) and decrease the formation of cholesterol^30^. Also, chromium can increase the activity of lecithin cholesterol acyltransferase (LCAT) and accelerate cholesterol esterification and excretion^31^,^32^. In addition, studies indicate that chromium activates glucose transporter glut4 through a cholesterol-dependent mechanism, which decreases cholesterol levels^33^,^34^,^35^.

Findings from the previous studies on chromium and fasting glucose levels have been controversial. A meta-analysis of randomized clinical trials (RCTs) found no significant effect of chromium supplementation on glucose concentrations as well as lipid profile in non-diabetic individuals, and was inconclusive with regard to glycaemia control among diabetic patients^36^. There are a few possible explanations for the inconsistent findings between the meta-analysis of RCTs and the present observational study. For example, chromium supplement may have different effect as compared to chromium derived from foods since chromium in food may have synergistic effects with other ingredients. Also, the dose of chromium in RCT is usually higher than that of typically found in food and the study duration in RCT is much shorter than that in the observational study. In addition, residual confounding in observational study cannot be excluded.

Our results concerning chromium and HOMA-IR are generally consistent with findings from two studies conducted in Korea, which found that hair chromium levels were inversely correlated with HOMA-IR^37^,^38^. Although the underlying mechanisms are not fully understood, studies indicate that chromium can increase the binding of insulin to cells and the number of insulin receptors as well as to activate insulin receptor kinase, which all lead to greater insulin sensitivity.

Several strengths enhanced the validity of our study. First, the study has a relatively large biracial sample and prospective design with a 23-year follow-up. Second, chromium levels in toenails were measured using INAA and toenail chromium is considered a reliable objective biomarker reflecting relatively long-term exposure^13^,^16^. Third, the observed inverse associations were robust, and remained after adjustment for a number of dietary and non-dietary factors including the baseline status of individual components of MetS that could potentially confound the association.

A few limitations need to be considered. First, toenail chromium was measured only a single time at baseline. Although toenail chromium can reflect a relatively long-term exposure, changes in chromium levels during the follow-up may affect the associations. Nevertheless, this effect is likely to be non-differential and may attenuate the true association between chromium and MetS and its components. Second, we could not differentiate trivalent chromium from hexavalent chromium in toenail measurement. Trivalent chromium is suggested to be beneficial and hexavalent chromium is toxic to human health^39^. Thus, the combination of these two forms may attenuate any association that may exist between trivalent chromium and MetS and its components. Nevertheless, no objective biomarkers of trivalent chromium are available for large-scale longitudinal study. Third, similar to any other observational study, the possibility of confounding from unknown or unmeasured factors cannot be completely excluded. Finally, the generalizability of our findings may be limited since participants were only recruited from urban areas of the US.

In conclusion, our results provide prospective human data showing that higher chromium levels in young adulthood are associated with lower incidence of MetS later in life. Specifically, chromium may have favorable effects on the lipid profile and the development of insulin resistance. Further studies are needed to establish causal inference and better understand the potential mechanism of action. Studies that separately examine the association of trivalent and hexavalent chromium with risk of MetS are also warranted.

## Additional Information

**How to cite this article**: Bai, J. *et al*. Chromium exposure and incidence of metabolic syndrome among American young adults over a 23-year follow-up: the CARDIA Trace Element Study. *Sci. Rep*. **5**, 15606; doi: 10.1038/srep15606 (2015).

## Figures and Tables

**Table 1:** Baseline characteristics of the study population by quartiles (Q) of toenail chromium levels, the CARDIA study, 1987 to 2010 (*n* = 3,648)*.

Characteristics	Total	Quartile of toenail chromium levels				*P*-value†
		Q1 (lowest)	Q2	Q3	Q4 (highest)	
No. of participants	3,648	912	912	912	912	
Toenail chromium, ppm	1.2 (2.3)	0.2 (0.1)	0.4 (0.1)	0.8 (0.2)	3.5 (3.7)	NA
Toenail mass, g	0.024 (0.013)	0.023 (0.13)	0.026 (0.014)	0.025 (0.013)	0.024 (0.013)	<0.01
Age, year	27.0 (3.6)	27.3 (3.5)	27.1 (3.6)	26.7 (3.7)	26.7 (3.7)	<0.01
Female, %	52.0	50.3	50.9	50.3	56.6	0.02
Black, %	48.0	46.7	50.6	47.6	47.0	0.32
Education, year	14.2 (2.4)	14.0 (2.3)	14.1 (2.3)	14.4 (2.5)	14.4 (2.5)	<0.01
Physical activity score, U	391.3 (290.5)	364.1 (273.3)	394.5 (299.6)	407.0 (299.8)	399.7 (287.1)	<0.01
BMI, kg/m^2^	24.8 (4.9)	24.8 (4.9)	25.1 (5.2)	24.7 (4.7)	24.7 (4.8)	0.16
Family history of diabetes, %	27.2	26.3	29.3	27.3	26.0	0.41
Smoking status, %						
Never	57.1	55.1	56.6	57.2	59.7	0.62
Former	13.9	14.1	14.6	13.9	12.9	
Current	29.0	30.8	28.8	28.8	27.4	
Alcohol, g/d	12.6 (22.9)	11.7 (24.7)	13.4 (24.2)	12.6 (21.0)	12.7 (21.5)	0.48
Triglycerides, mg/dL	75.6 (46.1)	78.0 (49.2)	76.2 (45.9)	76.6 (48.6)	71.5 (39.8)	0.02
HDL cholesterol, mg/dL	55.0 (13.8)	53.9 (13.7)	54.9 (13.8)	55.1 (13.5)	56.0 (14.0)	0.01
Glucose, mg/dL	82.0 (11.5)	82.3 (16.2)	81.6 (8.6)	82.6 (11.1)	81.3 (8.6)	0.054
Systolic blood pressure, mmHg	107.6 (10.4)	108.0 (10.1)	107.8 (10.8)	108.3 (10.5)	106.5 (10.2)	<0.01
Diastolic blood pressure, mmHg	67.5 (9.1)	67.7 (9.2)	67.8 (9.0)	67.6 (9.6)	66.8 (8.4)	0.08
Waist circumference, cm	79.2 (11.2)	79.4 (11.4)	79.7 (11.5)	79.1 (11.0)	78.5 (10.8)	0.14
LCn-3PUFAs, g/d	0.1 (0.2)	0.1 (0.2)	0.1 (0.2)	0.1 (0.1)	0.1 (0.2)	0.54
Magnesium, mg	405.6 (232.8)	402.6 (225.2)	411.1 (233.9)	413.2 (241.5)	395.7 (230.3)	0.35

Abbreviations: BMI, body mass index; CARDIA, Coronary Artery Risk Development in Young Adults; HDL, high-density lipoprotein; LCn-3PUFAs, long chain n-3 polyunsaturated fatty acids; NA, not applicable; Q, quartile. Thirteen out of 3648 participants had Cr levels under the limit of detection (LOD).

^*^Data are means (standard deviations), unless otherwise specified.

^†^*P*-values were for difference across quartiles of toenail chromium levels using analysis of variance, or chi-squared test.

**Table 2:** Multivariable-adjusted HRs (95% CIs) of metabolic syndrome by quartiles (Q) of toenail chromium levels, the CARDIA study, 1987 to 2010 (*n* = 3,648)*.

	Quartile of toenail chromium levels				*P* for linear trend†
	Q1 (lowest)	Q2	Q3	Q4(highest)	
Chromium- ppm	<0.266	0.266–0.551	0.552–1.225	≥1.226	
No. of participants	912	913	912	912	
No. of events	242	246	210	180	
Incidence rate-/1000 person years	15.01	14.95	12.63	10.59	
Model 1‡	1 (Referent)	1.02 (0.86–1.23)	0.89 (0.74–1.07)	0.76 (0.63–0.93)	0.002
Model 2§	1 (Referent)	0.97 (0.81–1.16)	0.87 (0.72–1.05)	0.74 (0.61–0.90)	0.003
Model 3||	1 (Referent)	0.97 (0.80–1.16)	0.88 (0.72–1.06)	0.80 (0.66–0.98)	0.006

Abbreviations: CARDIA, Coronary Artery Risk Development in Young Adults; CI, Confidence interval; HR, Hazard ratio; LCn-3PUFA, long chain n-3 polyunsaturated fatty acids; Q, quartile.

^*^All models were constructed using Cox proportional hazards regression analysis.

^†^*P* for trend was examined by using the continuous variable of toenail chromium levels.

^‡^Model 1: adjusted for age (years, continuous), sex, ethnicity (African American or Caucasian), and study center.

^§^Model 2: model 1 with additional adjustment for BMI(<18.5, 18.5–24.9, 25–30, ≥30 kg/m^2^), education(<12, ≥12, years), family history of diabetes(yes or no), smoking status(never smokers, former smokers, or current smokers), alcohol consumption(0,0.1–9.9,10.0–19.9, ≥20g/day), physical activity(quartiles), and intakes (quartiles) of LCn-3PUFA and magnesium.

^||^Model 3: model 2(except baseline body mass index, because it is highly correlated with waist circumference) with additional adjustment for individual components of metabolic syndrome at baseline.

**Table 3:** Multivariable-adjusted HRs (95% CIs) of incident components of the metabolic syndrome by quartiles (Q) of toenail chromium levels, the CARDIA study, 1987 to 2010*.

	Q1	Q2	Q3	Q4	*P* for trend†
Glucose (*n* = 3,992)					
Chromium- ppm	<0.265	0.265–0.549	0.550–1.215	≥1.216	
No. of participants	999	997	998	998	
No. of events	172	178	157	147	
Incidence rate/1000 person years	9.71	9.69	8.51	8.03	
Model 1‡	1 (Referent)	0.97 (0.79–1.20)	0.89 (0.71–1.11)	0.87 (0.70–1.10)	0.269
Model 2‡	1 (Referent)	0.88 (0.71–1.09)	0.87 (0.70–1.08)	0.85 (0.68–1.07)	0.376
Model 3§	1 (Referent)	0.91 (0.74–1.13)	0.85 (0.68–1.05)	0.87 (0.69–1.08)	0.395
Blood pressure (*n* = 3,647)					
Chromium- ppm	<0.266	0.266–0.551	0.552–1.232	≥1.233	
No. of participants	911	912	913	911	
No. of events	364	387	344	351	
Incidence rate/1000 person years	23.81	24.91	21.73	21.79	
Model 1‡	1 (Referent)	1.07 (0.92–1.23)	0.99 (0.85–1.15)	1.03 (0.89–1.20)	0.518
Model 2‡	1 (Referent)	1.03 (0.89–1.19)	0.98 (0.85–1.14)	1.05 (0.90–1.22)	0.750
Model 3§	1 (Referent)	1.07 (0.93–1.24)	0.96 (0.83–1.12)	1.08 (0.93–1.25)	0.667
Waist circumference (*n* = 3,536)					
Chromium- ppm	<0.263	0.263–0.550	0.551–1.231	≥1.232	
No. of participants	884	884	884	884	
No. of events	335	363	331	331	
Incidence rate/1000 person years	22.78	24.79	21.71	21.78	
Model 1‡	1 (Referent)	1.11 (0.95–1.29)	0.98 (0.84–1.14)	0.98 (0.84–1.15)	0.145
Model 2‡	1 (Referent)	1.15 (0.99–1.34)	0.98 (0.84–1.14)	0.94 (0.80–1.10)	0.234
Model 3§	1 (Referent)	1.23 (1.06–1.43)	1.07 (0.92–1.26)	1.09 (0.93–1.28)	0.674
Triglycerides (*n* = 3,563)					
Chromium- ppm	<0.266	0.266–0.553	0.554–1.232	≥1.234	
No. of participants	890	891	891	891	
No. of events	281	268	255	220	
Incidence rate/1000 person years	18.69	17.08	16.08	13.75	
Model 1‡	1 (Referent)	0.94 (0.79–1.11)	0.87 (0.73–1.03)	0.78 (0.65–0.94)	0.029
Model 2‡	1 (Referent)	0.96 (0.81–1.13)	0.88 (0.74–1.04)	0.79 (0.66–0.95)	0.040
Model 3§	1 (Referent)	0.97 (0.82–1.14)	0.85 (0.72–1.01)	0.82 (0.68–0.98)	0.045
HDL cholesterol (*n* = 2,989)					
Chromium- ppm	<0.269	0.269–0.561	0.562–1.244	≥1.245	
No. of participants	748	746	748	747	
No. of events	362	316	329	277	
Incidence rate/1000 person years	36.30	28.84	29.62	23.95	
Model 1‡	1 (Referent)	0.83 (0.71–0.97)	0.86 (0.74–0.997)	0.70 (0.60–0.83)	0.004
Model 2‡	1 (Referent)	0.83 (0.71–0.97)	0.87 (0.74–1.01)	0.70 (0.59–0.82)	0.003
Model 3§	1 (Referent)	0.84 (0.72–0.98)	0.93 (0.80–1.08)	0.75 (0.64–0.88)	0.030

Abbreviations: CARDIA, Coronary Artery Risk Development in Young Adults; CI, Confidence interval; HDL, high-density lipoprotein; HR, Hazard ratio; Q, quartile.

^*^All models were constructed using Cox proportional hazards regression analysis.

^†^*P* for trend was examined by using the continuous variable of toenail chromium levels.

^‡^Model 1 and model 2: adjusted for covariates cited in the footnote of Table 2.

^§^Model 3: model 2 with additional adjustment for the corresponding individual component at baseline.
